# Prospects for mineral biofortification of wheat: classical breeding and agronomy

**DOI:** 10.18699/vjgb-24-59

**Published:** 2024-09

**Authors:** I.N. Leonova, E.V. Ageeva, V.K. Shumny

**Affiliations:** Institute of Cytology and Genetics of the Siberian Branch of the Russian Academy of Sciences, Novosibirsk, Russia; Siberian Research Institute of Plant Production and Breeding – Branch of the Institute of Cytology and Genetics of the Siberian Branch of the Russian Academy of Sciences, Krasnoobsk, Novosibirsk region, Russia; Institute of Cytology and Genetics of the Siberian Branch of the Russian Academy of Sciences, Novosibirsk, Russia

**Keywords:** wheat, microelements, macroelements, breeding, agronomy, biofortification, пшеница, микроэлементы, макроэлементы, селекция, агрономия, биофортификация

## Abstract

Low intake of micro- and macroelements and vitamins in food negatively affects the health of more than two billion people around the world provoking chronic diseases. For the majority of the world’s population, these are soft and durum wheats that provide beneficial nutrients, however their modern high-yielding varieties have a significantly depleted grain mineral composition that have reduced mineral intake through food. Biofortification is a new research trend, whose main goal is to improve the nutritional qualities of agricultural crops using a set of classical (hybridization and selection) methods as well and the modern ones employing gene/QTL mapping, bioinformatic analysis, transgenesis, mutagenesis and genome editing. Using the classical breeding methods, biofortified varieties have been bred as a part of various international programs funded by HarvestPlus, CIMMYT, ICARDA. Despite the promise of transgenesis and genome editing, these labor-intensive methods require significant investments, so these technologies, when applied to wheat, are still at the development stage and cannot be applied routinely. In recent years, the interest in wheat biofortification has increased due to the advances in mapping genes and QTLs for agronomically important traits. The new markers obtained from wheat genome sequencing and application of bioinformatic methods (GWAS, meta-QTL analysis) has expanded our knowledge on the traits that determine the grain mineral concentration and has identified the key gene candidates. This review describes the current research on genetic biofortification of wheat in the world and in Russia and provides information on the use of cultivated and wild-relative germplasms to expand the genetic diversity of modern wheat varieties.

## Abstract (ru)

Недостаток потребления микро- и макроэлементов и витаминов в продуктах питания, который затрагивает более двух миллиардов человек на земном шаре, негативно сказывается на здоровье и приводит к развитию хронических заболеваний. Одним из источников полезных нутриентов является пшеница, которая обеспечивает пищевой энергией большинство населения мира. Создание современных высокоурожайных сортов привело к значительному обеднению минерального состава зерна и сокращению потребления минералов через продукты питания. Биофортификация – активно развивающееся направление, основной целью которого является улучшение питательных качеств сельскохозяйственных культур с помощью комплекса классических и современных методов. К числу основных технологий, используемых в программах биофортификации пшеницы, можно отнести традиционную селекцию, включающую методы гибридизации и отбора, современную селекцию с дополнительным привлечением методов картирования генов/QTL и биоинформатического анализа, трансгенез, мутагенез и геномное редактирование. Успехи в создании биообогащенных сортов были достигнуты в рамках различных международных программ, финансируемых HarvestPlus, CIMMYT, ICARDA, с помощью традиционной селекции и агрономических методов. Несмотря на перспективность методов трансгенеза и геномного редактирования для создания биообогащенных культур, они требуют значительных инвестиционных вложений и трудозатратны, поэтому данные технологии применительно к пшенице находятся в стадии разработки и не имеют пока практического выхода. В последние годы интерес к биообогащению пшеницы возрос в связи с успехами в области картирования генов и QTL для хозяйственно важных признаков. Разработка новых маркеров на основе результатов секвенирования генома пшеницы и привлечение биоинформатических методов анализа (GWAS, meta-QTL) расширили информацию по контролю признаков, определяющих содержание минералов в зерне, и выявили ключевые гены-кандидаты. В данном обзоре описано современное состояние исследований в области генетической биофортификации пшеницы в мире и в России. Приведены сведения об использовании гермоплазмы культурных и дикорастущих родственников для расширения генетического разнообразия современных сортов пшеницы.

## Introduction

As a source of complete plant protein, minerals, micro- and macronutrients and vitamins, wheat plays an important role for the world’s population. Consuming wheat products, the population obtains, on average, up to 20–30 % of calories per day; in some developing countries this figure is as high as 70 % (Shewry, 2009a; Shiferaw et al., 2013; Tadesse et al., 2019). To meet the growing demand for wheat grain, increasing yields has been the main focus for the breeders since the 1960s. Expansion of planted areas and introduction of new high-yielding varieties has gradually increased the world’s wheat production, so, according to the FAO, the grain harvest was estimated at 805.6 million tons in 2023 compared to less than 600 million tons in 2000 (https://www.fao.org/ worldfoodsituation/csdb/ru). Compared to then, significant yield increases of 1.3 to 1.8 times have been observed in major wheat-producing countries such as China, India, Russia and the United States (https://www.fao.org/faostat/ru/#country/).

However, this success in increasing wheat yields achieved through introduction of high-yielding varieties has been accompanied by deteriorated grain quality, reduced contents of protein, gluten and minerals that determine the nutritional value of the final product (Mitrofanova, Khakimova, 2017; Helguera et al., 2020). Published data indicate that the microand macronutrient contents in the grain of modern varieties have been significantly lower than those in ancient varieties and wild relatives (Salantur, Karaoğlu, 2021; Zeibig et al., 2022).

The micro- and macronutrients play an important role in many processes of plant development such as seed germination, root system development and yield formation (Marschner, 1995). They are also indispensable when it comes to photosynthesis and respiration and stress resistance regulation (De Santis et al., 2021; Shoormij et al., 2022; Khan et al., 2023). The list of macronutrients now considered essential for a healthy lifestyle and normal body function includes sodium (Na), potassium (K), magnesium (Mg), calcium (Ca), chlorine (Cl), phosphorus (P), and sulfur (S). Iron (Fe), zinc (Zn), copper (Cu), manganese (Mn), iodine (I), and selenium (Se) are commonly recognized as indispensable microelements (Jomova et al., 2022; Ali A.A.H., 2023). Some nutritionists additionally include bromine (Br), vanadium (V), silicon (Si), nickel (Ni) and chromium (Cr) on that list, but the data on the positive effects of these elements in animals and humans are currently contradictory (Vincent, 2017; Genchi et al., 2020).

Deficiencies in micronutrient intake with food, or the socalled “hidden hunger”, lead to the development of chronic diseases, reduced mental development and even increased mortality in most developing countries (Faber et al., 2014; Lockyer et al., 2018). Deficiency in Na, K, Ca, Mg, P results in nervous, cardiovascular, skeletal and muscular systems impairments. Among the essentials, these are the deficiencies of Fe, Zn, I and Se that are of particular concern, since they are involved in hemoglobin synthesis; regulation of the functions of a number of enzymes, including insulin; metabolism; cancer-cell suppression, etc. (Prashanth et al., 2015; Islam et al., 2023).

Currently, the improvement of wheat nutritional properties by increasing the concentration and bioavailability of essential micro- and macronutrients has become a priority in the field of wheat genetics and breeding. This direction commonly known as biofortification is developed through various approaches, the main ones being agronomic and genetic biofortification using both traditional breeding methods and modern molecular genetic approaches. The paper reviews the results obtained by the agronomic methods and classical breeding employing gene mapping technologies, and considers the prospects for their use in the development of biofortified wheat varieties.

## Mineral composition of wheat and its wild relatives

Mineral composition in wheat whole grains and flour varies a lot to be determined by the genotype, environments, soil composition, presence of mineral fertilizers and other agronomic factors. A significant contribution to the phenotypic manifestation and inheritance of the trait is made by the genotype, because it allows one to use samples with increased mineral content to breed new lines of wheat.

The mineral content in the grain of modern bread wheat varieties may change considerably: such elements as Zn, Fe, Cu, Mn range up to 40, 50, 4 and 38 μg/g, respectively; while the content K, Mg and Ca does not exceed 4,200, 1,150 and 370 μg/g on average (Murphy et al., 2008; Zhao et al., 2009; Khokhar et al., 2018; Morgounov et al., 2022; Potapova et al., 2023). Although today’s durum wheats do not differ significantly from soft ones in the concentration of major mineral elements (Ficco et al., 2009; Shewry et al., 2023), some authors indicate that Zn and Fe content in the grain of many durum wheat varieties has been significantly higher (Cakmak et al., 2010; Rachoń et al., 2012).

Modern wheat varieties have lower concentrations of macro- and micronutrients compared to their ancient, and wild and cultivated relatives. A number of studies have shown that the decrease in micronutrient content is not always related to changes in climatic factors or soil characteristics (Garvin et al., 2006; Ficco et al., 2009). M.S. Fan et al. (2008) conducted an extensive study of soil composition and the changes in Zn, Fe, Cu and Mn contents in wheat grains over 160 years. The mineral content was shown to have remained stable from 1845 to the 1960s, but then it declined significantly due to the introduction of yielding dwarfing varieties. This trend was maintained regardless of changes in the concentration of soil elements or the administration of organic and inorganic fertilizers. In other words, the reduced-height (Rht) genes in durum and soft wheat are accompanied by reduced micronutrient concentrations, the reduction level varies and depends on the genetic background of a variety (Velu et al., 2017a). Some authors note there are negative correlations between the yield of modern varieties and their Zn and Fe content. This may be the reason for the decrease in the concentration of the minerals in grain due to the cultivation of highly productive varieties (Monasterio, Graham, 2000; Garvin et al., 2006).

Breeding a biofortified wheat variety causes a problem of maintaining a high mineral content in the final products, since a significant part of micronutrients is concentrated in the grain shell, e. g., Zn, Fe and Cu concentrations reduce 2 to 10 times in flour if compared to that in whole grain, while in the bran made of grain hulls it remains several times higher (Peterson et al., 1986; Ciudad-Mulero et al., 2021). A good alternative may be using whole-wheat flour or adding bran that contains much more essentials into flour. Different proportions of wheat bran added into flour increase the Fe content in baked products, the greatest effect observed when adding 10 % of bran, which makes the bread comparable to that made of whole-wheat flour (Butt et al., 2004).

Screening the germplasms of wild and cultivated wheat relatives has also revealed significant differences in mineral concentrations. Despite the wide variability in Ca, Mg, K, Zn, Fe, Mn and Cu contents in the diploid and tetraploid ancestors of T. durum, T. dicoccum, T. monococcum, T. araraticum, T. timopheevii, and Ae. tauschii, scientists have observed that hexaploid wheat T. aestivum, on average, is inferior to them in the concentration of most elements (Marschner, 1995; Cakmak et al., 2004; Gupta P.K. et al., 2021; Zeibig et al., 2024). Zn and Fe concentrations in the grain of various representatives of the genus Aegilops (Ae. searsii, Ae. umbellulata, Ae. caudata, Ae. geniculata, etc.) are 2–3 times higher than those in modern hexaploid wheat cultivars (Gupta P.K. et al., 2021; Zeibig et al., 2022). High genetic diversity in relation to the mineral composition was found in wild spelt T. dicoccoides; a combination of high zinc, iron and protein contents in grain and high yield was also observed for some spelt varieties (Peleg et al., 2008; Chatzav et al., 2010).

Significant intra-population diversity is the basis for utilizing the genetic potential of wild and cultivated relatives as a source of high mineral content in grain for the presence of positive correlations between some element concentrations (Zn, Fe, Mg), protein content and yield allows for simultaneous improvement of several quality parameters without reducing productivity (Oury et al., 2006; Chatzav et al., 2010).

To improve the mineral composition, various introgression, addition, and substitution lines derived from hybridization of modern soft and durum wheat varieties with wild and cultivated relatives have been developed (Wang S. et al., 2011; Farkas et al., 2014; Savin et al., 2018). Examination of the given resources has enabled for identification of the accessions with better characteristics than the original commercial wheat varieties. It has also made it possible to detect the critical chromosomes containing targeted genetic factors to establish a basis for subsequent gene mapping.

An extensive source of genetic diversity for mineral composition in wheat are the synthetic hexaploid lines derived from the hybridization of different accessions of T. turgidum ssp. durum and Ae. tauschii (Alvarez, Guzmán, 2018; Morgounov et al., 2022). Using these synthetic lines, a large number of favorable target-gene alleles have been mapped that can be employed for genetic biofortification (Bhatta et al., 2018; Morgounov et al., 2022). However, a detailed analysis of the productivity of the accessions bred with these wheat relatives has shown that most of them are characterized by a decrease in yield and its components, depending on the genetic background of the recipient variety and the amount of alien genetic material (Calderini, Ortiz-Monasterio, 2003; Velu et al., 2017b), which significantly complicates the transfer of target genes into commercial wheat varieties.

## Genetic biofortification

Conventional breeding is the most common and cost-effective biofortification method to improve the mineral composition of wheat grain. In this classical method, donors of high nutrient content are crossed with a recipient variety possessing necessary economically important traits to select the sought trait in subsequent generations. If a foreign species is used as a donor, the process may be followed by several backcrossing cycles to transfer the targeted introgressed fragment and reduce the amount of foreign genetic material.

As a part of biofortification programs carried out in the major international centers involved in the study of cereal crops (CGIAR, CIMMYT, HarvestPlus, ICARDA), the results of screening of their collections of modern wheat varieties, landraces and wild species have been used to determine the mineral composition variability, develop recommendations and create pre-breeding lines. (Monasterio, Graham, 2000; Peleg et al., 2008; Ficco et al., 2009).

The use of traditional breeding methods for biofortification of wheat grain became a topical issue in Europe after the HEALTHGRAIN program was initiated (2005–2010) to summon 43 partners from 17 countries participated. Thanks to this program modern varieties and breeding lines, landraces of bread wheat and other cereal crops (rye, barley, oats) from European countries were evaluated for phytochemical components and mineral composition at several experimental plots. The results have shown that a large part of the trait variations was genetically determined, so the selected material may be available for breeding programs (Shewry, 2009b; Van Der Kamp et al., 2014).

Since 2003, HarvestPlus program has been investing heavily to develop biofortified varieties of wheat, rice, corn, millet, beans, sweet potato and other crops with higher levels of vitamin A, Fe and Zn. Their wheat biofortification program is underway in Africa (Egypt, Ethiopia, Madagascar, Nigeria, South Africa, Zambia and Zimbabwe), Asia (Afghanistan, Bangladesh, China, India, Nepal, Pakistan, the Philippines) and Latin America (Bolivia, Brazil, Mexico) (https://www. harvestplus.org/biofortification-hub). To date, under the program, 37 biofortified wheat varieties for countries in Asia and Africa have been developed, of which 12 are high-yielding and resistant to fungal diseases (Andersson et al., 2017; Bouis, Saltzman, 2017; Kamble et al., 2022). The study of Znbiofortified varieties developed in India under the HarvestPlus brought the authors of the experiment to a conclusion that despite the low contribution of genotype to the overall variability of Zn concentration in grain, the biofortified genotypes exhibited environmental stability when grown in different soil types, including those with low Zn content (Khokhar et al., 2018). A list of biofortified soft and durum wheats developed by major breeding institutions in India, Pakistan, Bangladesh, Nepal and Bolivia in collaboration with CIMMYT and recommended for commercial cultivation is presented in Gupta O.P. et al. (2022). These include durum wheat varieties HI8777 and MACS 4028 with Fe content of 48.7 and 46.1 mg/kg and Zn content of 40.3 and 43.6 mg/ kg, respectively; and soft wheat varieties WB 02, HI 1633, DBW 187, DBW 332 and PBW 757, whose concentration of these elements exceeds 40 mg/kg.

In recent years, close attention has been paid to the development of biofortified wheat genotypes of non-standard grain color (blue, purple, black) that differ from conventional redgrain and white-grain varieties by a high content of anthocyanins having antioxidant, antimicrobial and anticarcinogenic activity. Investigation of the pigmented samples has shown that some of them have additional characteristics such as increased protein and micro- and macronutrient content (Sharma S. et al., 2018; Xia et al., 2020; Dhua et al., 2021; Liu Y. et al., 2021). Analysis of the flour made of blue, green and black grains has found that the pigmented varieties exceed the standard ones in protein and amino acid content by 7–18 %, while their zinc content is almost 2-fold higher, and that of Fe and Mn varies from 8 to 40 % (Tian et al., 2018). There is also evidence that the blue-grain wheat has higher iron and zinc contents if compared to those of purple, red and white varieties (Ficco et al., 2014). Experiments to search for samples with high Se content that has antitumor activity have been conducted for the pigmented wheats (Xia et al., 2020). They demonstrated that when the plants were sprayed with Se or when the last was applied to soil, the purple-grain varieties accumulated more Se in grain if compared to the white-grain ones. However, according to other authors, in the absence of additional selenium treatments, the blue- and purple-grain varieties were inferior to standard wheat varieties by almost 5 times (Phuong et al., 2017).

Pigmented wheats may contain increased amounts of gluten, anthocyanin and minerals, and for that reason they are considered as a promising source of useful nutrients for bakery and pasta products. However, the studies having investigated the detailed qualitative characteristics of the final products made of pigmented-wheat flour are few and include mainly the assessment of anthocyanins, protein and gluten content, dough characterization and a description of organoleptic properties (Pasqualone et al., 2015; Vasilova et al., 2021; Sharma N. et al., 2022; Fitileva, Sibikeev, 2023; Gordeeva et al., 2023). Nevertheless, encouraging results proving that such products retain significantly more beneficial nutrients while processing have already been obtained (Padhy et al., 2022). For example, A. Kumari et al. (2020) analyzing the chapati baked from pigmented wheat varieties showed that the wheat samples ranged in the following descending order in terms of their phenolic content, anthocyanins and antioxidant activity: black > blue > purple > white grain. Currently, colored-grain varieties are considered as a promising source of bioactive substances and high antioxidant activity.

## Quantitative trait loci mapping

An important stage of biofortification is the selection of potential genotypes containing target loci, whose presence leads to an increase in mineral elements in grain. Currently, it is DNA markers being used for nearly three decades for mapping quantitative trait loci (QTL) and for marker-assisted and genomic selection (Collard, Mackill, 2008). That is of great importance, since a detected QTL localization and position on a chromosome allows one to understand the genetic basis of a trait, identify the loci controlling mineral-elements content as well as new QTLs and, based on the information obtained, select the genotypes suitable for breeding.

Two approaches are used to localize target loci and identify new gene alleles: genetic mapping on the populations raised from biparental crosses, and genome-wide association study (GWAS), whose main advantage is the use of the genotype panels characterized by high genetic diversity (Collard, Mackill, 2008; Tibbs Cortes et al., 2021).

In the last 15 years, a sufficient number of papers have been published on mapping of the QTLs whose presence determines essentials content in wheat grain. It should be noted that most of these studies have been conducted mainly to identify genomic regions controlling Zn and Fe concentrations, as these elements are considered indispensable for human health (Peleg et al., 2009; Tiwari et al., 2009; Wang S. et al., 2011; Hao et al., 2014; Pu et al., 2014). GWAS has enabled for more accurate mapping of the genomic loci, so new previously unpublished QTLs have been identified, and functional candidate genes have been searched for in the regions of target loci (Bhatta et al., 2018; Alomari et al., 2019; Rathan et al., 2022; Tadesse et al., 2023). Comprehensive research to identify the key genomic regions for Zn and Fe biofortification in soft wheat was conducted by P. Juliana et al. (2022), who, using a panel of 5,585 advanced-generation pre-breeding lines, identified 141 markers on all wheat chromosomes except for 3A and 7D. The results summarizing the QTL localizations for Zn and Fe contents in wheat grain are presented in part in review articles (Garcia-Oliveira et al., 2018; Gupta O.P. et al., 2022). Currently, researchers are accumulating information on the most informative loci, their localization and validating SNP-KASP markers. So far, only the first steps have been taken towards developing the KASP markers based on the mapped QTLs (Wang Y. et al., 2021; Sun M. et al., 2023) and there is no available information on their specificity and practical use.

Only a limited number of studies cover the issue of genetic and association mapping of the QTLs responsible for other mineral elements (Alomari et al., 2017; Manickavelu et al., 2017; Wang P. et al., 2017; Qiao et al., 2021; Hao et al., 2024). Comparative genomic and meta-QTL analyses identified more than 400 stable loci for some of which pleiotropic effects were shown in relation to different mineral elements and yield components (Shariatipour et al., 2021; Singh et al., 2022; Potapova et al., 2023; Cabas-Lühmann et al., 2024). A GWAS conducted for 205 winter soft wheat genotypes from China revealed more than 280 marker-trait associations with Ca, Mn, Cu, and Se contents in different wheat chromosomes. The study also demonstrated that the gene clusters in chromosomes 3B and 5A (for Ca), 4B (for Cu), and 1B (for Mn) had the highest contribution to their content (Wang W. et al., 2021). Based on a whole-genome analysis of 252 soft wheat cultivars for Se content, it was concluded that the use of the SNP markers linked to target loci in chromosomes 5D and 1D could increase Se concentration by 6.62 % during genomic selection (Tadesse et al., 2023). A GWAS performed on a panel of 768 cultivars found the genomic regions associated with Cu, Fe, K, Mg, Mn, P, Se, and Zn concentrations in soft wheat and the stably expressed candidate genes located in the QTL localization regions (Hao et al., 2024). Eleven loci associated with calcium accumulation were detected in chromosomes 2A, 3A (2 loci), 3B (2 loci), 3D, 4A, 4B, 5B (2 loci), and 6A, of which four QTLs were stably expressed under different environmental conditions (Shi X. et al., 2022). Candidate-gene study by these authors identified the TraesCS4A02G428900 gene in chromosome 4A, whose high expression may be associated with calcium accumulation in wheat grains.

To find sources and donors of the efficient loci associated with high mineral concentrations in grain, a search for new loci was conducted using various bread wheat relatives and synthetic hexaploid wheats (SHWs) (see the Table). The SHWs obtained from crosses between tetraploid species (T. durum, T. dicoccum) and diploid Ae. tauschii have become a source of new gene alleles for various agronomically important traits. According to Z.E. Pu et al. (2014), 22 of the 29 alleles responsible for increased concentration of Zn, Fe, Mn, Cu and Se in the grain of recombinant inbred lines originate from the genome of a synthetic line derived from the crossing of T. turgidum ssp. turgidum and Ae. tauschii ssp. tauschii. A significant number of loci, including novel ones, have been identified in the genome-D chromosomes originating from different varieties of Ae. tauschii, which demonstrates the high potential of this species in increasing the content of such elements as Ca, Co, Cu, Li, Mg, Mn and Ni in grain (Bhatta et al., 2018; Krishnappa et al., 2021; Morgounov et al., 2022).

A number of studies have shown that the presence of foreign chromosomes in the genomes of substitution, introgression and addition wheat lines leads to increased concentrations of Zn, Fe, and other minerals (Wang S. et al., 2011; Velu et al., 2017c; Gupta P.K. et al., 2020; Potapova et al., 2023). In diploid wheat species (T. monococcum, T. boeoticum), two loci responsible for Fe content were identified in chromosomes 2A and 7A and one responsible for Zn in chromosome 7A (Tiwari et al., 2009; see the Table). In different populations of cultivated and wild tetraploid species, recombinant inbred and synthetic lines have had many QTLs originating from the A and B genomes of T. durum, T. dicoccum, and T. dicoccoides and associated with Fe and Zn content (Peleg et al., 2009; Crespo-Herrera et al., 2016, 2017; Cabas-Lühmann et al., 2024). It is noteworthy that a number of mapped QTLs for Zn, Fe, Mn, and other minerals have no negative effects on grain protein content, 1,000-grain weight, and yield in general, which allows one to improve these varieties for several traits simultaneously (Uauy et al., 2006; Liu J. et al., 2021; Cabas-Lühmann et al., 2024). Also, many studies have shown a high level of heritability of the studied traits that indicates the genotype’s significant contribution. It will make it possible to use the samples with foreign translocations as a sour

**Table 1.**
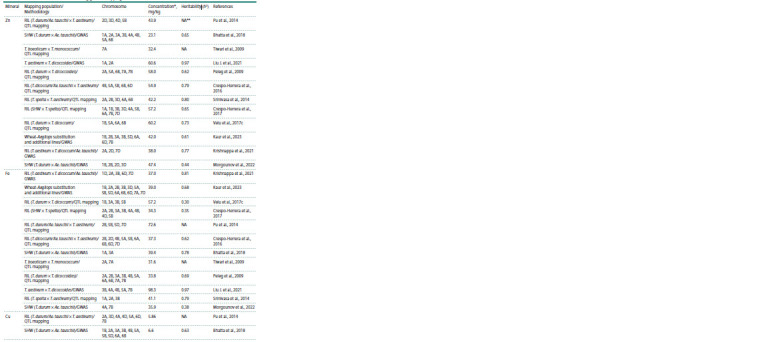
Chromosomal localization of the loci associated with grain mineral content in synthetic hexaploid lines and wheat relatives that were detected using genetic mapping and GWAS * Average values of field estimations; ** no data available (NA).

**Table 1end.**
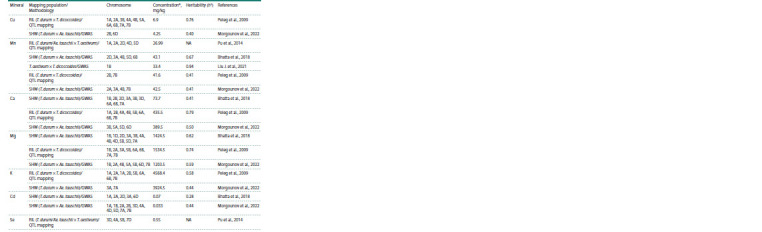
Table 1end

## Agronomic methods

The simplest and most accessible of all biofortification methods is employment of the fertilizers enriched with micro- and macroelements that either applied to the soil or as a foliar treatment. Several studies suggest that applying different concentrations of nitrogen fertilizer alone or in combination with mineral supplements can have a positive effect on grain micronutrient content (Shi R. et al., 2010; Kutman et al., 2011; Niyigaba et al., 2019). As for the effectiveness of different methods (seed treatment, soil fertilization, and foliar spraying) in terms of their effects on the yield, protein content, and mineral concentration, foliar spraying has so far been regarded as the most effective one (Stepien, Wojtkowiak, 2016; Hassan et al., 2019; Saquee et al., 2023), e. g., the efficacy analysis of foliar spraying of wheat with Zn fertilizers conducted on 23 experimental fields in seven countries (China, India, Kazakhstan, Mexico, Pakistan, Turkey, and Zambia) showed an 80–90 % increase of Zn concentration in grain and no reduction in yield (Zou et al., 2012).

Many authors consider Zn solutions for foliage fertilizing an important tool for ensuring proper zinc concentrations in vegetative tissues during grain filling that increases Zn concentration in grain (Cakmak et al., 2010; Velu et al., 2014). The efficacy of such a treatment was demonstrated in experiments on Se biofortification of durum wheat (De Vita et al., 2017). According to the authors, Se concentration after grinding as well as in pasta increased by 11 %, while there was no decrease in other quality indicators, such as yield and pasta organoleptic characteristics

It is important to point out that the data on the effects different fertilizers have on the mineral-substance concentrations in grain are quite contradictory. Some researchers have noted a lack of correlations between fertilizer application and mineral accumulation in grain due to complex interactions of several factors such as environmental conditions, genotype, fertilizer application rates, mechanized tillage, etc. (Jaskulska et al., 2018; Caldelas et al., 2023).

The main disadvantages of agronomic biofortification are the fertilizers have to be applied every season; and one has to take into account a number of additional factors such as soil structure, the amounts of essentials concentrated in it, lack or excess of precipitation, temperature conditions, biological uptake degree, and genotype influence (Kostin et al., 2020). According to I. Cakmak et al (2010), lack of adequate moisture level, high soil pH, high CaCO3 content and low organic-matter concentration significantly reduce the availability and uptake of Zn and Fe from the soil, which prevents their optimum concentration in grain

Another direction of agronomic biofortification is using soil microorganisms (Bacillus, Azotobacter, Acinetobacter, Pseudomonas, Rhizobium, etc.) for solubilization of mineral substances to enhance their mobility from soil to edible plant parts. It has been shown that seed inoculation or application of microorganisms directly into the soil lead to an increase in the concentration of such elements as Zn, Fe, Mn, Cu and Se in wheat grain and shoots (Rana et al., 2012; Golubkina et al., 2017; Sun Z. et al., 2021; Ali M. et al., 2023). The mechanisms of microbial biofortification and the efficiency of the method for Fe and Zn uptake in various agricultural plants has been described in a review by S. Verma et al. (2021).

Apart from rhizosphere microorganisms, researchers have also experimented with arbuscular mycorrhizal fungi as an additional agent to improve the agronomically important traits of crops. The strains, either alone or in combination with rhizosphere microorganisms, increases the concentration of macronutrients (N and P), micronutrients (Zn and Fe) in wheat grain as well as wheat productivity parameters (1,000-grain weight; number of grains per ear; and number of productive tillers) (Ma et al., 2019; Yadav et al., 2020).

Despite the encouraging results obtained, a limited success has been achieved so far in this field due to the complexity of the interaction mechanisms between the microorganisms and the host plant and the influence of abiotic environmental factors such as soil mineral composition, temperature, and phytic acid effect on Zn and Fe bioavailability. The efficiency of microbial biofortification also significantly depends on the genotype, suggesting additional experiments are to be caried out to assess genotype responsiveness and select effective microbial strains (Garg et al., 2018).

## Biofortification in Russia

Russia has seen practically no studies into wheat varieties to create the genotypes with increased content of mineral elements. To date, only limited data have been published on screening of domestic varieties and breeding lines for some micro- and macronutrients content and on the development of technologies for the use of fertilizers, growth regulators and microorganisms to improve the mineral composition of grain (Golubkina et al., 2017; Aristarkhov et al., 2018; Chikishev et al., 2020), e. g., Institute of Biology of the Karelian Scientific Center of RAS has been devising techniques for increasing Cu content in the root and shoots of Triticum aestivum L. and Hordeum vulgare L. (Kaznina et al., 2022).

As a part of Comprehensive Kazakh-Siberian Program under the Central Asia Sustainable Innovation Bureau (CASIB) umbrella, new varieties and breeding lines have been regularly screened for yield, grain, flour and baking qualities. As for the mineral composition, works in this area have just begun and the first data on the analysis of large collections of hexaploid wheat varieties and synthetic lines of different geographical origins have been published (Shamanin et al., 2021; Morgounov et al., 2022). The grains of the Russian varieties investigated under the CASIB program had higher Zn content than the varieties developed under the HarvestPlus program (Shamanin et al., 2021; Shepelev et al., 2022). However, the Russian-Kazakhstani samples were inferior to the genotypes from the USA and Japan in terms of Fe, Ca, Mo and Mg content.

Apart from breeding the varieties promising for functional nutrition, studies have been performed to produce purplegrained wheat. The presence of anthocyanins has shown not to affect the technological properties of bread, and adding purple-grain bran into flour has enriched bakery products with dietary fiber and anthocyanins (Fisenko et al., 2020). Nadira, a purple-grain variety of spring soft wheat is distinguished by increased antioxidant activity, disease resistance and high yield (Vasilova et al., 2021).

Studies have been initiated to identify genetic factors and map genes/QTLs in varieties of Russian origin as well as in synthetic, recombinant and introgression wheat lines (Morgounov et al., 2022; Potapova et al., 2023). First steps have been taken to develop genomic breeding models to improve the mineral composition of wheat grain (Potapova et al., 2024).

## Conclusion

Biofortification is one of the modern and effective approaches aimed at enriching wheat grain with essential vitamins and minerals. Not only does it help to overcome the mineral elements deficiency in grain, but also to improve grain quality, yield and resistance to many diseases. The biofortification programs devised for the creation of new wheat genotypes with improved properties use different approaches, the main being traditional breeding that employs modern technologies of genetic mapping and agronomic techniques.

As for transgenesis and genomic editing, these technologies are still under development and have no current practical application. Genetic biofortification is considered to be more economically efficient and has a longer validity period than agronomic one. At present, the search for promising sources and donors for improving the mineral composition of wheat grain is supposed to be conducted in several directions: 1) study of variability of micro- and macronutrient concentrations among the ancient wheat varieties having greater genetic diversity if compared to modern ones; 2) search for new genetic loci in the germplasm of wheat relatives and creation of target gene donors with their participation; 3) development and use of the new DNA markers based on cereal genome sequencing data; 4) improvement of the gene/QTLs mapping methods employing bioinformatic approaches to identify the key candidate genes associated with mineral accumulation; 5) development of genomic breeding programs for targeted creation of biofortified genotypes. These methods of genetic fortification combined with optimal agro-technological methods will allow us to solve the problem of mineral nutrients deficiency in food

## Conflict of interest

The authors declare no conflict of interest.
